# HIV-Sheltering Platelets From Immunological Non-Responders Induce a Dysfunctional Glycolytic CD4^+^ T-Cell Profile

**DOI:** 10.3389/fimmu.2021.781923

**Published:** 2022-02-11

**Authors:** Aiwei Zhu, Fernando Real, Jaja Zhu, Ségolène Greffe, Pierre de Truchis, Elisabeth Rouveix, Morgane Bomsel, Claude Capron

**Affiliations:** ^1^ Mucosal Entry of HIV and Mucosal Immunity, Institut Cochin, Université de Paris, Paris, France; ^2^ Institut National de la Santé et de la Recherche Médicale (INSERM) U1016, Paris, France; ^3^ Centre National de la Recherche Scientifique (CNRS) UMR8104, Paris, France; ^4^ Service d’Hématologie, Hôpital Ambroise Paré (AP-HP), Boulogne-Billancourt, France; ^5^ Université Versailles Saint Quentin-en-Yvelines (UVSQ), Université Paris Saclay, Versailles, France; ^6^ Service d’Infectiologie, Hôpital Raymond Poincaré (AP-HP), Garches, France

**Keywords:** HIV-1, immunological failure, platelets, CD4^+^ T-cell metabolism, glycolysis, virus-containing platelets

## Abstract

Immunological non-responders (InRs) are HIV-infected individuals in whom the administration of combination antiretroviral therapy (cART), although successful in suppressing viral replication, cannot properly reconstitute patient circulating CD4^+^ T-cell number to immunocompetent levels. The causes for this immunological failure remain elusive, and no therapeutic strategy is available to restore a proper CD4^+^ T-cell immune response in these individuals. We have recently demonstrated that platelets harboring infectious HIV are a hallmark of InR, and we now report on a causal connection between HIV-containing platelets and T-cell dysfunctions. We show here that *in vivo*, platelet–T-cell conjugates are more frequent among CD4^+^ T cells in InRs displaying HIV-containing platelets (<350 CD4^+^ T cells/μl blood for >1 year) as compared with healthy donors or immunological responders (IRs; >350 CD4^+^ T cells/μl). This contact between platelet containing HIV and T cell in the conjugates is not infectious for CD4^+^ T cells, as coculture of platelets from InRs containing HIV with healthy donor CD4^+^ T cells fails to propagate infection to CD4^+^ T cells. In contrast, when macrophages are the target of platelets containing HIV from InRs, macrophages become infected. Differential transcriptomic analyses comparing InR and IR CD4^+^ T cells reveal an upregulation of genes involved in both aerobic and anaerobic glycolysis in CD4^+^ T cells from InR vs. IR individuals. Accordingly, InR platelets containing HIV induce a dysfunctional increase in glycolysis-mediated energy production in CD4^+^ T cells as compared with T cells cocultured with IR platelets devoid of virus. In contrast, macrophage metabolism is not affected by platelet contact. Altogether, this brief report demonstrates a direct causal link between presence of HIV in platelets and T-cell dysfunctions typical of InR, contributing to devise a platelet-targeted therapy for improving immune reconstitution in these individuals.

## Introduction

Approximately 20% of the overall combination antiretroviral therapy (cART)-treated patients are immunological non-responders (InRs) who fail to reconstitute a competent immune status despite prolonged viral suppression as a result of proper treatment observance (1). The causes of this immunological failure remain unclear, and no treatment is available to improve CD4^+^ T-cell count restoration and health of InRs (1), who are at higher risk of AIDS and non-AIDS morbidity and mortality (2–4).

Using blood obtained from virally suppressed HIV-infected patients under cART (viral load below detection limit for >1 year prior to sampling date, here referred to as cART-suppressed individuals), we have recently shown that platelets from InRs specifically carry infectious HIV *in vivo* (5). Hence, platelets from cART-suppressed individuals carry infectious HIV regardless of patient viremia and platelet numbers but are significantly correlated with CD4^+^ T-cell nadir (<200 cells/μl) and sustained low blood CD4^+^ T-cell counts (<350 CD4^+^ T cells/μl) (5). Predictive statistical analyses indicated that the chance of remaining with CD4^+^ T-cell count <350 CD4^+^ T cells/μl in the next 18 months despite cART is >50-fold higher in individuals with platelets sheltering HIV than in individuals without HIV in platelets (5). These results indicate that the presence of HIV in platelets is a characteristic of InRs. However, a direct causal relationship between the presence of HIV in platelets and immunological failure remains elusive.

The poor immunological recovery in InR is mainly driven by a sustained low CD4^+^ T-cell count that relies on persistent inflammation and immune activation affecting T-cell population profiles (6–10). Platelets may have a role in this process. Indeed, platelet–CD4^+^ T-cell conjugates have been observed in the peripheral blood of patients with autoimmune disease, suggesting that platelets may be involved in regulating T-cell activation (11). Moreover, platelets form an increased proportion of conjugates with T cells in peripheral blood from HIV-infected patients compared to healthy controls (12). Platelets express adhesive proteins that not only promote platelet aggregation responsible for primary hemostasis (clotting) but also mediate interactions with leukocytes such as monocytes/macrophages or T cells. In particular, platelets can drive inhibition of proliferation and differentiation of either CD4^+^ T cells into regulatory profiles (FoxP3^+^ T_reg_) (13) or Th17 in chronic inflammation (14). As platelet–T-cell conjugates form in the blood of HIV patients (12), we therefore speculate that HIV-containing platelets would directly act on CD4^+^ T cells, causing T-cell dysfunction. Furthermore, polarization of T cells to activated or resting states requires changes in metabolism that might be implicated in InR immunological failure (15).

Here, in this brief report, we approached platelet-mediated T-cell dysfunction *in vitro* by characterizing comparatively the T-cell metabolic changes induced by HIV-containing platelets from InRs vs. immunological responders (IRs). In contrast with IR platelets, HIV-containing platelets from InR individuals induced an increased production of energy *via* glycolysis in CD4^+^ T cells, not macrophages. This platelet-mediated glycolytic stimulus is not related to a platelet-mediated T cell infection, as HIV-containing platelets do not infect T cells upon *in vitro* interaction in contrast to macrophages. This is to our knowledge the first evidence of a direct causative action of HIV-containing platelets on T-cell dysfunctions.

## Materials and Methods

### Ethics Statement

This non-interventional study was approved by the institutional review board of the “Comité de Protection des Personnes” (CPP) of Ile-de-France (VIH-PLAQUETTES, ID-RCB: 2020-A00307-32) and conforms to the principles outlined in the Declaration of Helsinki. Accordingly, all participants were informed in writing about the study and allowed not to participate.

### Patient Sample Preparation

Platelet-rich plasma (PRP) and peripheral blood mononuclear cell (PBMC) samples were obtained during routine blood testing of 32 HIV-infected individuals on cART from the French Hospital Database on HIV receiving care at the Ambroise Paré (Boulogne-Billancourt, France) and Raymond Poincaré (Garches, France) Hospitals. The enrollment criterions for the French Hospital Database on HIV were confirmed HIV-1 and cART initiated for at least 1 year before the time of blood sampling. Human PBMC and PRP samples from healthy HIV-seronegative donors used as a negative control for all experiments were obtained from the French blood collection center [Etablissement Francais du Sang (EFS) Paris, France]. PRP and PBMCs were prepared as previously described (5).

InR subjects were defined as having sustained CD4^+^ T-cell counts below 350 cells/μl for more than 6 months during viral suppression, i.e., plasma viral load copies below limit of detection (LOD) as detected by the Abbott RealTime HIV-1 assay on an automated m2000 system. IR subjects were defined as having sustained CD4^+^ T-cell counts above 350 cells/μl during virological suppression during this time. Clinical information on InR and IR subjects is presented in **Table 1**.

**Table 1:** Clinical data and HIV detection in platelets from InR and IR individuals included in the study.

Individuals information		InR	IR	Statistical test
	Total of HIV-infected individuals on cART	n = 20	n = 12	-
	Biological sex (male/female number)	15/5	10/2	ns, *p* = 0.304, chi-square
	Age [mean years (IQR)]	51 (41–67)	46 (30–51)	ns, *p* = 0.659, Mann–Whitney
	Years since HIV diagnosisis [mean years (IQR)]	14 (7–19)	13 (4–22)	ns, *p* = 0.904, Mann–Whitney
	Months with confirmed status (responder or non-responder) [mean months (IQR)]	39 (10–56)	39 (31–82)	ns, *p* = 0.794, Mann–Whitney
	Months with undetectable viral load [mean months (IQR)]	38 (16–56)	34 (24–78)	ns, *p* = 0.754, Mann–Whitney
				
**Clinical parameters**		**InR**	**IR**	**Statistical test**
	Platelet count [mean million platelets per ml (IQR)]	209 (148–292)	218 (206–358)	ns, *p* = 0.704, Mann–Whitney
	CD4+ T-cell count [mean cells per ml (IQR)]	221 (165–327)	923 (815–1,292)	*p < 0.001, Mann–Whitney
	Total lymphocyte count [mean million cells per ml (IQR)]	1.54 (1.3–1.9)	2.5 (2.0–2.8)	*p < 0.001, Mann–Whitney
	HIV+ platelets (FISH-flow) (positive/negative number)*	12/3	3/9	*p = 0.004, chi-square
	HIV+ platelets (FISH-flow) [mean per million platelets in positive group (IQR)]**	1,113 (120–1,220)	150 (135–160)	*p = 0.003, Mann–Whitney

*number of individuals positive or negative for HIV in platelets as detected by FISH-flow method.

**mean number with IQR of HIV+ platelets per million platelets in individuals detected positive for HIV in platelets.

IQR: interquartile ranges showing 25th and 75th percentiles of data.

HIV, human immunodeficiency virus; InR, Immunological non-responders; IR, Immunological responders; cART, combination antiretroviral therapy; FISH-Flow, fluorescent in situ hybridation; IQR, Interquartile range.

### Flow Cytometry

#### Peripheral Blood Mononuclear Cell

**Figure 1:**
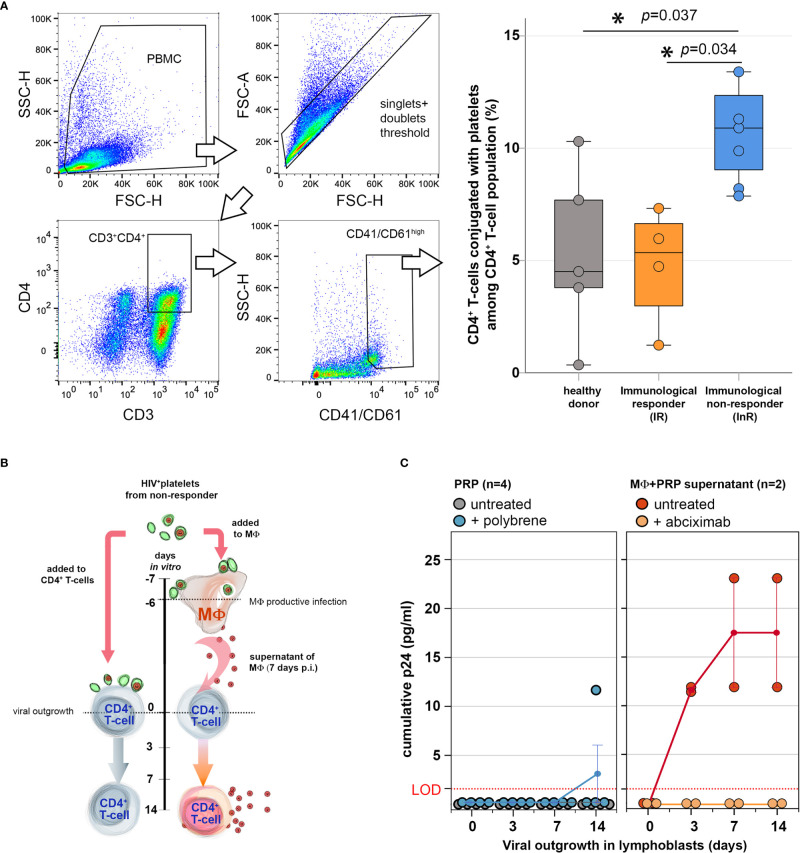
Platelets from immunological non-responders (InRs) form more conjugates with CD4^+^ T cells *in vivo* but do not transfer infectious virus to CD4^+^ T cells *in vitro*. **(A)** Platelet–CD4^+^ T-cell conjugates are more frequent in the circulation of InRs *in vivo*. Flow cytometry analysis of CD4^+^ T cells (CD3^+^CD4^+^) conjugated with platelets (CD41/61^high^) circulating in the blood of combination antiretroviral therapy (cART)-treated patients virally suppressed. Left: Gating strategy to assess platelet–T-cell conjugates. Right: Percentage of platelet–CD4^+^ T cell (CD3^+^CD4^+^CD41/61^high^) conjugates among CD4^+^ T cells (CD3^+^CD4^+^) as detected in healthy donor (gray) and HIV-infected patient samples categorized as immunological responder (IR, orange) and InR (blue). Kruskal–Wallis, statistically relevant differences *p* < 0.05 (*). **(B, C)** Upon direct interaction, HIV-containing (HIV^+^) platelets propagate infection to macrophages (MΦ), not to CD4^+^ T cells. **(B)** *In vitro* experimental setup. Left: HIV^+^ platelets are directly incubated with activated CD4^+^ T cells from healthy donors for a 14-day viral outgrowth assay. Right: MΦ from heathy donors interacts overnight with HIV^+^ platelets. At day 7, MΦ culture supernatants are collected and tested for their infectious replicative virus content by addition of the same activated CD4^+^ T cells from healthy donors as in left for a 14-day viral outgrowth assay. **(C)** Viral production quantification: HIV-1 is quantified in coculture supernatants after 14 days of viral outgrowth in CD4^+^ T cells that were incubated directly with HIV^+^ platelets in the presence or absence of polybrene (left) or incubated with supernatants of MΦ infected upon interaction with HIV^+^ platelets in the presence or absence of abciximab (right). LOD, limit of detection for the technique. *Statistically significant.

Multiparametric flow cytometry was performed using frozen PBMCs collected from InR, IR, and healthy donors. PBMCs were thawed, washed two times in phosphate buffered saline (PBS) with 2% fetal bovine serum (FBS), and resuspended in PBS supplemented with 2% FBS containing antibodies for surface marker staining at 1:20 v/v concentration each (CD4-PerCP and CD3-APC from BD Biosciences; CD41/61-PE A2A9/6 clone from BioLegend) for 15 min at room temperature. Next, cells were washed in PBS two times, fixed in 4% paraformaldehyde (PFA; EuroMedex) for 30 min, and washed three times before proceeding to flow cytometry data acquisition in a GUAVA 12HT system. The gating strategy for assessing CD4^+^ T cell–platelet conjugates was performed by selecting cells and not debris based on forward (FSC) and side scatter (SSC), limited inclusion of doublets by FSC-area/FSC-height strategy, gating CD4^+^ T cells by CD4-PerCP^+^ and CD3-APC^+^ double-positive events, and finally gating CD41/61-PE^high^ cells among CD4^+^ T-cell population (**Figure 1A**).

#### Platelet-Rich Plasma

InR and IR platelets were tested for the presence of HIV using the Fluorescence In Situ Hybridisation (FISH)-flow method we described earlier (5), which comprises double detection of HIV RNA and HIV p24 capsid protein in CD41/61-positive platelets quantified by flow cytometry. Briefly, platelets were fixed in PFA 4% for 30 min at room temperature and immunostained for CD41/61 and p24 before *in situ* hybridization with HIV Gag mRNA probes designed with the Stellaris Probe Designer program (https://www.biosearchtech.com/support/tools/design-software/stellaris-probe-designer) as we described (5). In our previous study (5), InR platelet samples in which HIV could not be detected by this methodology represent 22% of total InR samples, whereas IR platelets positive for HIV represent 14% of total IR samples. To be able to evaluate the role of the presence of HIV in platelets in both IRs and InRs, we thus selected for this study a set of 15 samples from InRs from which 12 were positive and 3 were negative for HIV and 12 samples from IRs from which 3 were positive and 9 were negative for HIV. HIV-containing platelets that we refer to below correspond to the population of platelets in which around 0.1% (5) of platelets do actually contain the virus and not to platelets that all individually contain HIV, unless otherwise stated.

### Platelet–Leukocyte *In Vitro* Cocultures

#### Platelet–CD4^+^ T-Cell Cocultures

CD4^+^ T-cells from healthy donors were purified by negative selection from healthy donor’s PBMCs using the EasySep Negative Human CD4 Kit (STEMCELL Technologies). A pool of cells from three independent donors were used for each experiment. CD4^+^ T cells from healthy donors were cultivated in RPMI 1640 (Gibco) supplemented with 10% FBS, L-glutamine (2 mM, Gibco), and penicillin/streptomycin (100 U/ml, Gibco) at 10^6^ cells/ml and activated by adding 2.5 μg/ml of phytohemagglutinin-L (PHA-L from *Phaseolus vulgaris*, Sigma-Aldrich) for 48 h. After two washes in RPMI 1640, CD4^+^ T cells were resuspended in fresh medium without or with platelets from InRs, IRs, or healthy donors, respectively, and cultivated overnight at 37°C prior to processing for downstream experiments. The platelet:T cell ratio employed was 2:1.

#### Platelet–Macrophage Coculture

Monocytes from healthy donors were purified by negative selection from PBMCs from healthy donors using the EasySep Human Monocyte Enrichment Kit (STEMCELL Technologies). For each experiment, a pool of cells from three independent donors was used. Monocytes were differentiated into M2-like macrophages by cultivation in RPMI 1640 supplemented with 10% FBS in the presence of macrophage colony-stimulating factor (M-CSF; 25 μg/ml, Sigma-Aldrich) for 6 days, followed by M2-like macrophage polarization using interleukins IL-4 and IL-13 (20 ng/ml each) for 2 days as described (5). The macrophages were incubated with platelets from InRs, IRs, or healthy donors overnight prior to processing for downstream experiments. To account for the cell size differences between T cells and macrophages, the platelet:macrophage ratio employed was 5:1 (5).

### Viral Outgrowth Assay

To measure comparatively infectious viral production from CD4^+^ T cells and macrophages, we adapted our previously described VOA using CD4^+^ T cells from healthy donors as reporter cells.

#### HIV Transfer From Platelet to CD4^+^ T Cells

Healthy donor’s CD4^+^ T cells were activated by PHA-L (5 μg/ml) for 48 h and cultured in RPMI medium supplemented with 10% FBS and IL-2 at 20 U/ml (complete medium) for an additional 48 h before use in the VOA. To evaluate infectious viral production after platelet–CD4^+^ T-cell interaction, HIV-containing platelets from InRs were added to 10^5^ activated healthy donor’s CD4^+^ T cells cultured in complete medium with or without 20 μg/ml polybrene to facilitate fusion of viral envelope and host cell membrane (16). The platelet:T cell ratio employed was 2:1. After 7 days of coculture, medium was replaced and freshly prepared 10^5^ healthy donor’s activated CD4^+^ T cells were added to the T cells that interacted with HIV-containing platelets to improve viral propagation for an additional 7 days. At days 0, 3, 7, and 14 of the VOA assay, culture medium was collected and replaced by fresh medium. Collected media were analyzed by HIV p24 capsid protein ELISA (Innotest HIV Antigen mAb, FUJIREBIO) to measure the cumulative viral production.

#### HIV Transfer From Platelets to Macrophages

In parallel experiments, to quantify viral outgrowth by macrophages upon interaction with InR platelets, macrophages were cocultured with HIV-containing platelets from InRs for 7 days in the presence or absence of the anti-platelet agent abciximab (ReoPro, Janssen Inc.) as described (5). Next, the macrophage–platelet coculture medium was collected and centrifuged, the supernatants were added for a 14-day period to the same pools of healthy donor’s CD4^+^ T cells used above for evaluating HIV transfer from platelets to CD4^+^ T cells, and viral outgrowth was quantified as above.

### Metabolic Profiling

Real-time cell metabolic analysis was carried out by Seahorse XFp Analyzer using the ATP Rate Assay Kit (Agilent) and following manufacturer’s instructions. Platelets from InRs, IRs, or healthy donors were cultivated at 37°C overnight with healthy donor’s CD4^+^ T cells or macrophages (pool of three donors, 5 × 10^5^ cells/well) in 48-well plates or in Seahorse XFp cell culture plates, respectively. After thorough wash out of unconjugated platelets, cell metabolism was further analyzed. The platelet:leukocyte ratio employed was 2:1 and 5:1 for CD4^+^ T cells and macrophages, respectively. For CD4^+^ T cells that had interacted with platelets, T cells were centrifuged at 300 g for 10 min, resuspended in Seahorse XF RPMI medium, and placed into Seahorse XFp cell culture plates pretreated with Cell-Tak (Corning, Fisher Scientific Inc.) following manufacturer’s instructions. For macrophages that interacted with platelets directly in the Seahorse XFp cell culture plates, complete medium was replaced by Seahorse XF RPMI medium. The ATP Rate assay protocol established for the Seahorse XFp Analyzer by the manufacturer provides dynamic information on the total ATP energy production by mitochondrial respiration *via* oxidative phosphorylation (OXPHOS) and/or by glycolysis in living cells. Basic oxygen consumption rate (OCR), which indicates aerobic respiration by OXPHOS, and extracellular acidification rate (ECAR), which indicates glycolysis, were measured over 1 h at indicated time points before and after successive addition of OXPHOS inhibitor oligomycin and the mitochondrial respiratory inhibitors rotenone/antimycin. Results were analyzed by the Agilent Seahorse Analytics online software (seahorseanalytics.agilent.com) to assess the levels of OCR (pmol/min), ECAR (mpH/min), and ATP production rate (pmol/min). A metabolic cell energy map was built by correlating OCR vs. ECAR values, displaying data in a spectrum of metabolic profiles comprising aerobic (predominant ATP production *via* mitochondrial respiration), glycolytic (predominant ATP production *via* glycolysis), quiescent (low ATP production rate by both pathways), or energetic (high ATP production rate by both pathways). Results were displayed as mean with standard errors of values obtained from biological replicates of platelet samples (InR n = 6; IR n = 5; and healthy donors n = 6).

### Microarray Data Analysis

The public microarray dataset GSE106792 from Gene Expression Omnibus (GEO) comparing the gene expression of CD4^+^ T cells from InRs, IRs, and healthy donors (17) was reassessed using the GEO2R tool provided by the GEO website. Datasets were grouped in InRs, IRs, and healthy donors according to the Immune Non-Responders, Immune Responders, and Healthy Donors donor class metadata, respectively, but disregarding the source name metadata related to CD71 expression. Dimensional reduction was performed by Uniform Manifold Approximation and Projection (UMAP) of all individual datasets (from InRs, IRs, and healthy donors) as provided by GEO2R software. Confidence ellipses were plotted on UMAP in R software using UMAP coordinates. Volcano plots were generated in SPSS software (IBM) using the data output of the volcano plots comparing InR vs. IR gene expression generated by GEO2R software. The genes most significantly upregulated in InRs vs. IRs in the differentially expressed gene (DEG) analysis were selected based on Log_2_ fold change >0.5 and the -Log_10_ *p* value >3. The lists of DEG in InRs vs. IRs were used for biological pathway enrichment analysis and visualization of the biological processes enriched in InRs vs. IRs as described (18).

These lists were also submitted to g:Pprofiler (https://biit.cs.ut.ee/gprofiler/gost) (19). The statistical tests were performed by the g:GOSt functional profiling tool, and *p* values were calculated as described in https://biit.cs.ut.ee/gprofiler/page/docs, “section significance threshold”. Briefly, the tool uses an in-house algorithm called g:SCS that performs multiple testing corrections outperforming the commonly used Bonferroni correction (BC) or Benjamini–Hochberg False Discovery Rate (FDR).

The graphical representation of the pathway enrichment analysis was performed by applying g:Profiler output into a Cytoscape Enrichment Map as described (18). Nodes represent gene sets that participate in a given function, and their size represents the number of genes composing this functional cluster. Lines connecting nodes indicate an overlap between gene sets composing both connected nodes. The line width represents the number of genes common to both nodes.

Next, the list of DEGs in InRs vs. IRs was submitted to HumanCyc metabolic map software (20) to visualize the contribution of DEGs on different metabolic pathways in InRs vs. IRs.

### Statistical Analysis

All statistical analyses were performed using SPSS software (IBM) and parametric and non-parametric tests for normal and non-normal data distributions, respectively. Statistical significance is indicated by asterisks in figures and was established by *p* values <0.05.

## Results

### *In Vivo* Platelets from Immunological Non-Responders (InRs) Form more Conjugates With CD4+ T Cells as Compared With Immunological Responder (IR) Ones

To address the immunomodulatory role of platelets on CD4^+^ T cells in immunological failure, we first investigated the presence of platelet–CD4^+^ T-cell conjugates in PBMCs from InRs, IRs, and healthy donors by flow cytometry. The median frequency of platelet–CD4^+^ T-cell conjugates among CD4^+^ T cells was 11% in InRs as compared with 6% in IRs and 3.5% in healthy controls (**Figure 1A**), indicating that platelets from InRs form more conjugates with CD4^+^ T cells than platelets from IRs *in vivo* that could result in deleterious effects to T cell functions.

### Platelets from InRs do not Transfer Infectious Virus to CD4+ T Cells

The InR subjects tested for platelet–CD4^+^ T-cell conjugates harbor HIV in platelets as quantified by FISH-flow cytometry (see *Materials and Methods*), in contrast to IR subjects whose platelets were negative for viral components. A consequence of the intimate contact between HIV-containing platelets from InR and T cells when conjugates formed could be the transfer of HIV productive infection from platelet to T cells and in turn in their production of infectious viruses. To measure these infectious viruses, we established a viral outgrowth assay (VOA) in which HIV-containing platelets from InRs were allowed to interact directly with healthy donor CD4^+^ T cells or macrophages. Resulting productive infection of CD4^+^ T cells or macrophages was then quantified (**Figure 1B**). No outgrowth was detected in healthy donor’s CD4^+^ T cells (serving also as infection reporter cells) after interaction with HIV-containing platelets from InRs, even in the presence of polybrene, a facilitator of membrane fusion (**Figure 1C**, left). In contrast, we observed that the transfer of HIV sheltered in InR platelets to macrophages is productive, as infection spreads replication-competent virus to healthy donor’s reporter CD4^+^ T cells (**Figure 1C**, right). Adding the anti-GPIIbIIIa platelet drug abciximab to the platelet–macrophage coculture blocked this platelet-mediated HIV transfer to macrophages *in vitro*, as we demonstrated previously (5). Thus, unlike macrophages, infectious HIV enclosed in InR platelets does not target CD4^+^ T cells, although HIV-containing platelets might immunomodulate CD4^+^ T-cell functions, in turn triggering the immunological failure observed in non-responders.

### CD4+ T Cells from InR are More Prone to Aerobic Glycolysis as Compared with IR Ones

**Figure 2:**
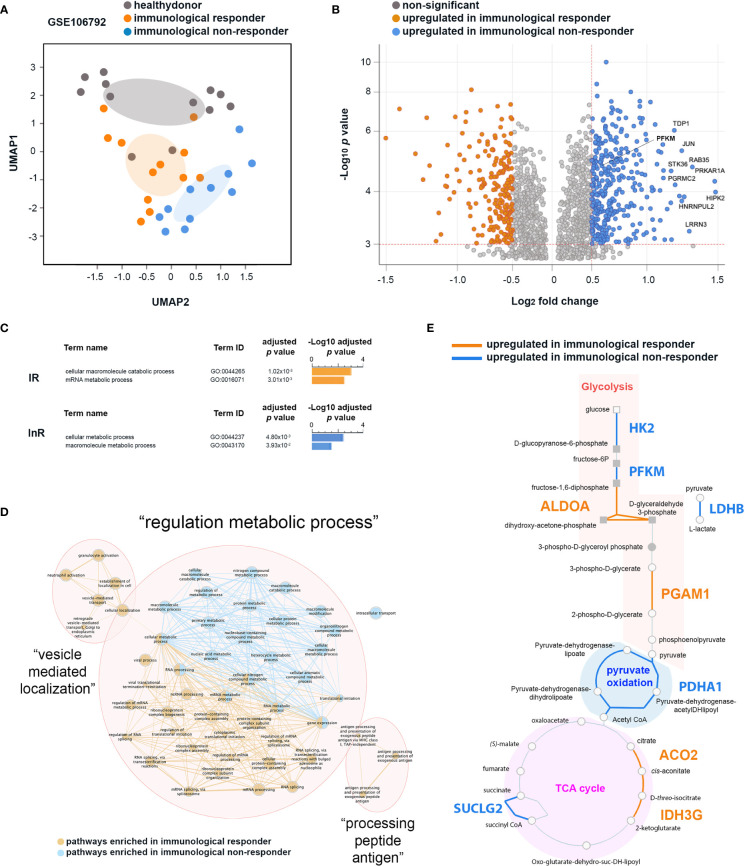
Differential transcriptomic analyses of CD4^+^ T cells from immunological non-responder (InR) and immunological responder (IR) patients focusing on genes associated with metabolic pathways. **(A)** InR, IR, and healthy donor transcriptomes have distinct profiles. Uniform Manifold Approximation and Projection (UMAP) of transcriptomic data from the GSE106792 dataset based on CD4^+^ T cells InR, IR, and healthy donors grouping individuals according to blood CD4^+^ T-cell counts and assessed by the Gene Expression Omnibus (GEO) constructed at a probability of 0.99. **(B)** Volcano plot from the differentially expressed gene (DEG) analysis between InR and IR CD4^+^ T cells. In the *y* axis, significance is indicated by red dotted line threshold at -Log_10_ *p* value >3 for both InR and IR datasets. In the *x* axis, significance is indicated by red dotted line thresholds at Log_2_ fold change >0.5 for InR and <-0.5 for IR DEGs. **(C)** Differential biological pathway analysis activated in InR vs. IR CD4^+^ T cells using g:Profiler. **(D)** Network of biological pathways enriched in CD4^+^ T cells from InR (blue nodes and connectors) or from IR (orange nodes and connectors) **(E)** HumanCyC metabolic map showing genes upregulated in CD4^+^ T cells from InR (blue font) and upregulated in CD4^+^ T cells from IR (orange font) implicated in glycolysis, pyruvate oxidation, and TCA cycle biochemical reactions.

Different studies have demonstrated the role of cellular metabolism in the innate and adaptive host responses to infection (21), especially in T-cell immunity (22, 23). To get insight on the CD4^+^ T cell functional changes induced by InR platelets, we reassessed a transcriptome dataset obtained by Younes et al. (17), reporting on different transcriptome signatures presented by InR, IR, and healthy donor CD4^+^ T cells. We now used an improved analytical pipeline comprising also metabolic pathway networks (18, 20). Our revised grouping of InR, IR, and healthy donor samples relied only on the provided metadata of clinical status but discarding the CD71 marker used to substratify InR samples as performed in the original analysis (17). Transcriptomic data of InR, IR, and healthy donor as we grouped and compared in a dimensional reduction strategy showed that differences between the three groups remained conserved (**Figure 2A**).

We then thus focused our differential analysis on two major metabolic pathways implicated in T-cell activated vs. resting states: OXPHOS and glycolysis (24). Among the genes upregulated in InR CD4^+^ T cells as compared with IR counterparts in a DEG analysis, a significant number was implicated in glycolytic pathways (**Figure 2B**). Upregulated genes in InRs included Tyrosyl-DNA phosphodiesterase 1 (TDP1) that decreases phosphoglycolate formation, and phosphoglycolate is known to have an effect on two steps in glycolysis (25); Leucine Rich Repeat Neuronal 3 (LRRN3), a clinically relevant biomarker of immune status in HIV-1 infection whose upregulation correlates with a less senescent T-cell phenotype (26), that binds to LEDGF/p75 or LEDGF/p52 involved in HIV-1 integration (27) and associates with glycolysis canonical gene products ENO2, HK2, PFKFB3; Phosphofructokinase muscle type (PFKM) that is a subunit of phosphofructokinase enzyme, a central controller of the mammalian glycolytic pathway (28); Homeodomain-interacting protein kinase-2 (HIPK-2) that is a member of a family of proteins upregulated in highly proliferative tumors whose expression correlates with upregulation of genes involved in aerobic glycolysis (29); Progesterone Receptor Membrane Component protein-2 (PGRMC2) that is increased in glycolysis (30); and finally, STK36 that plays an important role in the Sonic hedgehog (SHH) pathway that regulates the activity of GLI transcription factors and drives glucose metabolism (31, 32). When analyzed together for functional enrichment using g:Profiler (19), genes specifically upregulated in InRs contributed to two main biological pathways about metabolism, namely, cellular metabolic processes (GO:0044237) and macromolecule metabolic pathways (GO:0043170), whereas those increased in IRs concern macromolecule catabolic processes (GO:0044265) and mRNA metabolic processes (GO:0016071) (**Figure 2C**).

Next, to get a better insight into how these biological pathways interact together, we built a network of biological processes most significantly enriched in CD4^+^ T cells from InRs as compared with those from IRs. Three main networks of biological processes emerged: “vesicle mediated localization”, “processing peptide antigen”, and “regulation metabolic process” (**Figure 2D**). The “regulation metabolic process” cluster connects biological pathways enriched in both InR and IR CD4^+^ T cells. However and in agreement with g:Profiler biological pathway analyses, whereas the nodes connecting biological pathway in IR CD4^+^ T cells relate mainly to mRNA processing, those connecting biological pathways in InR CD4^+^ T cells are mainly composed of macromolecule metabolic processes connected with pathways involved in ATP production. To better define the genes upregulated in InR CD4^+^ T cells directly implicated in ATP production, we submitted the list of DEGs in InR vs. IR T cells to the HumanCyc database (20) to build a metabolic map. This map showed which enzymes that are implicated in biochemical reactions leading to ATP production would be differentially regulated in IR and InR CD4^+^ T cells. Although both sets of cells harbor differentially upregulated genes for enzymes involved in glycolysis, pyruvate oxidation, and Tricarboxylic Acid TCA cycle, InR CD4^+^ T cells show a remarkable upregulation of genes coding for enzymes involved in specific reactions of the glycolytic pathway (**Figure 2E**). Notably, Hexokinase 2 (HK2) is responsible for breaking glucose in D-glucopyranose-6-phosphate, ATP-dependent 6-phosphofructokinase muscle type (PFKM) is responsible for breaking fructose-6P in fructose-1,6-diphosphate, and, very importantly, Lactate Dehydrogenase B (LDHB) is responsible for breaking pyruvate (the end product of glycolysis) into L-lactate. This last reaction is a hallmark of aerobic glycolysis, a metabolic reaction performed by activated effector T cells (33). The upregulation of LDHB gene in InR as compared with IR CD4^+^ T cells indicates that InR CD4^+^ T cells have increased aerobic glycolysis or Warburg effect, a characteristic of T-cell activation states (34). Altogether, the analysis of CD4^+^ T-cell transcriptomic data indicates that InR CD4^+^ T cells are more prone to aerobic glycolysis than their IR counterparts, but the potential role of increased platelet–T-cell conjugates we observed in InRs in this process could not be assessed.

### HIV-Containing Platelets from InRs Stimulate Energy Production in CD4+ T Cells via Glycolysis in CD4+ T Cells, not in Macrophages

**Figure 3:**
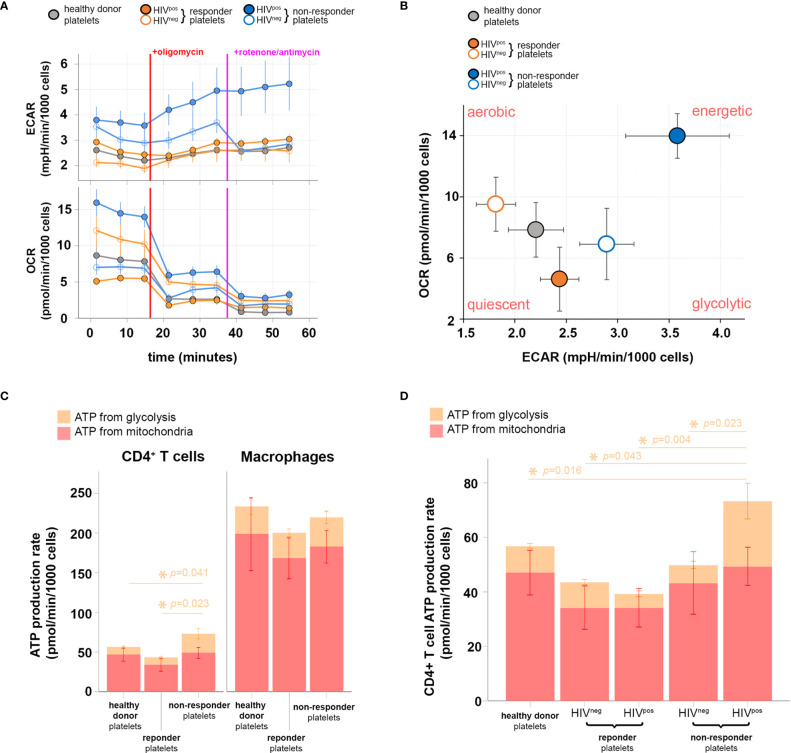
Metabolic profiling of CD4^+^ T cells after interaction with immunological non-responder (InR), immunological responder (IR), or healthy donor platelets, sheltering or not HIV in platelets. **(A)** Oxygen consumption rate (OCR; upper) and extracellular acidification rate (ECAR; lower) obtained throughout 1 h of data acquisition from live CD4^+^ T cells that interacted with InR (blue), IR (orange), or healthy donor (gray) platelets. Filled or empty circles with the same color code indicate the presence (HIV^pos^) or absence (HIV^neg^) of HIV in platelets, respectively. During the time course of data acquisition, the oxidative phosphorylation (OXPHOS) inhibitor and mitochondrial respiratory chain blocker oligomycin and rotenone/antimycin were injected at time points indicated by red and purple lines, respectively. **(B)** Energy phenotype map of CD4^+^ T cells that interacted with InR (blue), IR (orange), or healthy donor (gray) platelets. Filled or empty circles with the same color code indicate the presence (HIV^pos^) or absence (HIV^neg^) of HIV in platelets, respectively. **(C)** ATP production rate from CD4^+^ T cells (left) or macrophages (right) that interacted with InR, IR, or healthy donor platelets. The contribution of either glycolysis or mitochondria respiration to ATP production is discriminated in the bar graph by orange and red, respectively. Asterisks indicate statistically relevant differences established using a threshold of *p* < 0.05 calculated with a Kruskal–Wallis test on glycolysis levels (orange). Mitochondrial ATP production does not differ between groups using the same statistical test and significance threshold. **(D)** ATP production rate from CD4^+^ T cells that interacted with healthy donor platelets, IR platelets without HIV (HIV^neg^) in platelets, IR platelets with HIV (HIV^pos^) in platelets, InR HIV^neg^ platelets, and InR HIV^pos^ platelets. The contribution of either glycolysis or mitochondria respiration to ATP production is discriminated in the bar graph by orange and red, respectively. Asterisks indicate statistically relevant differences established by threshold of *p* < 0.05 using ANOVA on glycolysis levels (orange). Mitochondrial ATP production does not differ between groups using the same statistical test and significance threshold.

To approach this question experimentally, we cocultured InR, IR, or healthy donor platelets with healthy donor CD4^+^ T cells and evaluated the platelet-induced metabolic profile of these T cells. Accordingly, glycolysis and mitochondrial respiration in CD4^+^ T cells after coculture with platelets were evaluated simultaneously and in live cells by measuring ECAR and OCR, respectively. CD4^+^ T cells were sequentially treated with oligomycin (ATP synthase and OXPHOS inhibitor) and antimycin A/rotenone (mitochondrial complex III and complex I inhibitors) to functionally assess OXPHOS and mitochondrial respiration capacities. InR platelets positive for the presence of HIV in platelets induced in CD4^+^ T cells a glycolysis baseline increase compared with InR lacking HIV in platelets, IR (positive or negative of HIV in platelets), or healthy donor platelets (**Figure 3A**, upper graph). No difference was observed in mitochondrial respiration baseline induced by platelets from the five studied conditions with the exception of InR platelets positive for the presence of HIV in platelets, capable of inducing an increased baseline mitochondrial respiration (**Figure 3A**, lower graph). As expected in this system, when the mitochondrial inhibitors were added sequentially, the mitochondrial respiration was drastically inhibited in all groups, and a compensatory increase in glycolysis was observed in CD4^+^ T cells that was remarkable and more prominent after interaction with InR platelets positive for the presence of HIV as compared with all the other conditions (**Figure 3A**).

By correlating mitochondrial respiration capacities with the glycolytic compensatory increase, we built a bioenergetics phenotype map for CD4^+^ T cells that interacted with InR or IR platelets, either positive or negative for HIV in platelets, or healthy donor platelets. In contrast with InR platelets without HIV, IR platelets positive or negative for HIV or healthy donor platelets, in which ATP production relies mostly on mitochondrial respiration (aerobic profile), InR platelets positive for HIV induce a more energetic phenotype in CD4^+^ T cells, in which the ATP production is promoted by both mitochondrial respiration and glycolysis, indicating aerobic glycolysis (**Figure 3B**).

We then compared the contribution of mitochondrial respiration and glycolysis to ATP production in CD4^+^ T cells vs. macrophages after interaction with InR platelets positive for HIV, IR platelets negative for HIV, or healthy donor platelets using the same dynamic metabolic measurements. Macrophages inherently produce more ATP than CD4^+^ T cells upon interaction with all platelets tested, with macrophage ATP being produced mostly by mitochondrial respiration. In contrast, an augmented ATP production resulting mainly from an increase in ATP generated by glycolysis was observed in CD4^+^ T cells that interacted with InR compared with IR or healthy donor platelets (**Figure 3C**).

We next compared specifically the ATP production *via* glycolysis induced in CD4^+^ T cells after interaction with InR or IR platelets, positive or negative for HIV, or healthy donor platelets. In contrast to the other experimental conditions in which ATP production *via* glycolysis is similar whatever the type of platelets they had interacted with, interaction between InR platelets positive for HIV and CD4^+^ T cells resulted in higher ATP production *via* glycolysis compared with interaction with IR platelets, independently of the presence of HIV, or healthy donor platelets (**Figure 3D**).

## Discussion

Although HIV infection is evolving into a chronic condition with comparable life expectancy to the uninfected population in patients treated with ART (35, 36), an increased risk remains in ART-treated individuals of developing serious non-AIDS complications despite viral suppression (37–41). However, these non-AIDS comorbidities are associated with low CD4^+^ T-cell levels (42) and are thus frequent in InRs—unable to restore proper CD4^+^ T-cell levels—that represent 20% of HIV-infected individuals (1, 3, 8, 43). There are currently no available treatments to improve InR immune recovery (8, 44). Chronic immune activation could be in part responsible of these non-AIDS comorbidities, but the exact mechanism of this phenomenon is not totally elucidated (11, 45, 46). It is thus important to define parameters associated with immunological failure in CD4^+^ T cells from InRs at the cellular and molecular level.

We have recently shown that platelets from InRs can shelter HIV in direct correlation with immune failure (5), but the mechanism behind this correlation remains unclear. Accumulating evidence indicates that platelets can modulate lymphocyte functions (47). Indeed, besides hemostasis, human platelets also carry important immunological functions (48). Thus, platelets can participate in the pathology of the disease during chronic inflammatory conditions such as atherosclerosis, sepsis, and rheumatoid arthritis (49–51).

We now report that one of the mechanisms by which platelets contribute to immunological failure might rely on the capacity of platelets from InRs compared to IRs to form increased conjugate numbers with CD4^+^ T cells *in vivo*, as also observed in the blood of HIV patients (12). HIV-containing platelet–T-cell conjugates do not result in HIV transfer to CD4^+^ T cells, in contrast to macrophages in which HIV-containing platelets can propagate infection *in vitro* (5).

This result contrasts with another study by Simpson et al. (52) showing that CD4^+^ T cells are permissive to HIV carried by platelets. However, the clinical status of patients studied in this publication differs from our own study, as samples were obtained from patients before cART administration that were thus viremic and from patients treated for 3 months by cART with a majority of patents remaining viremic. Consequently, platelets used in this publication had the capacity to bind and uptake circulating HIV and transfer infection to T cells *via* viruses attached to their surface. Furthermore, the mean patient CD4^+^ T-cell counts was >460 cells/μl, indicating that the majority of samples studied by Simpson et al. (52) fall outside the group of individuals that most frequently have HIV in platelets [CD4^+^ T-cell count of <350 cells/μl as described (5)].

In our study, by contrast, individuals were all cART-treated for more than 6 months before sampling, sustainedly aviremic (blood viral load below level of detection) and the InR group had sustained low CD4 counts of <350 cells/μl for >6 months, similar to our previous study (5). Accordingly, and as we have shown previously, the platelets studied here did not harbor cell-free HIV attached to their surface and the virus is only found inside platelet intracellular compartments. Furthermore, as no cell-free virus was circulating in the patients we have studied here, their platelets did not capture virus at their surface nor endocytosed it from the blood, as we already demonstrated (5), hampering for example a platelet-mediated T-cell infection by viruses carried on the surface of platelets. Thus, the mechanism of virus transfer from platelets to CD4^+^ T cells likely occurs only by synaptic-like transfer during conjugate formation whereby the virus exits platelet internal compartments to reach and infect T cells.

We cannot exclude that increasing the platelet:T cell ratio in our *in vitro* coculture system would effectively promote platelet-mediated T-cell infection as observed by Simpson et al. (52) using platelets from viremic patients. However, our results demonstrate that HIV-containing platelets interacting with T cells at a low platelet:T cell ratio are sufficient to cause T-cell dysfunction even if this ratio does not promote CD4^+^ T infection.

Accordingly, independent of infection, InR platelets containing HIV can induce a change in CD4^+^ T-cell metabolism resulting in augmented glycolysis. This effect is specific to CD4^+^ T cells interacting with InR platelets that contain HIV, as we found that CD4^+^ T cells that interacted with platelets from InR lacking HIV, IR (containing or not HIV), or from healthy donors lack these capacities, and macrophages are insensitive to platelet-induced metabolic changes.

The energetic increase in T-cell metabolism induced by InR platelets we report could participate in chronic immune CD4^+^ T-cell activation and in turn CD4^+^ T-cell exhaustion as in other pathologies (11, 45, 46). Furthermore, the higher level of T-cell immune activation during chronic HIV infection (12) may enhance the formation of these conjugates and in turn maintain a state of chronic inflammation by increasing T-cell trafficking through inflamed tissues harboring HIV replication (12).

Increased glycolysis is a well-defined mark of T-cell activation (53). We show that platelets from InRs in which virus can be detected activate CD4^+^ T cells by increasing CD4^+^ T cell glycolysis in aerobic conditions. We hypothesize that the metabolic state of CD4^+^ T cells activated upon conjugate formation with HIV-containing platelets has two main implications: 1) these CD4^+^ T cells become exhausted, inducing an immunosenescence scenario related to immunological failure; and 2) these CD4^+^ T cells might reverse pro-viral latency and stimulate the production of new viral particles despite the ART. This reactivation would in turn maintain an “active CD4^+^ T-cell reservoir” (54) producing low-level, but constant, viral mRNA and potentially persistent viral particles despite ART. Validation of both scenarios will require further experiments.

The interaction of platelets with lymphocytes results in inhibition of T-cell proliferation and drives the differentiation of naive or memory CD4^+^ T cells toward regulatory profiles (T_reg_ : FoxP3^+^) or inflammatory ones like Th17 thus resulting in immunological failure (13, 55). It is still to be determined whether this pathway regulates *in situ* T-cell function, as suggested for patients with rheumatoid arthritis, leading to inefficient viral elimination and perpetuating inflammation (11). Along this line, the pro-inflammatory environment in InR individuals is linked to circulating T_reg_/Th17 unbalance accompanied by their functional dysregulation (17, 56, 57).

Several mechanisms have been proposed to account for immunomodulation resulting from platelet interaction with CD4^+^ T cells. Platelets can directly interact with lymphocytes by direct contact, inducing their polarization and/or secretion of cytokines/chemokines (58). Platelets can also shed microvesicles (ectosomes) that directly contact these lymphocytes (55) as well as myeloid and epithelial cells (47, 48, 58). Functionally, these platelet microvesicles could transfer active mRNA and microRNA (miRNA) to target cells (58, 59) and could promote T_reg_ differentiation (55).

The platelet–CD4^+^ T-cell conjugates we quantified here rely on the identification of platelets by CD41/CD61. We cannot exclude the possibility that part of, or all of, CD4^+^ T cells might have conjugated with platelet microvesicles. Additional morphological analyses would be required to solve this issue, at least qualitatively.

Platelet microvesicles can use multiple mechanisms to exert these effects such as extracellular signaling through receptors following transient interaction, transfer of surface molecules by trogocytosis-like mechanism, and delivery of their content including RNA and miRNA to the target cell cytoplasm (58, 59), thereby promoting platelet-derived mRNA by target T cells.

In particular, platelets contain a repertoire of mainly pro-inflammatory miRNAs such as miRNA-155 and miRNA-326, involved in nuclear factor (NF)-κB-mediated inflammatory macrophage responses (60) and Th17 cell polarization (61–63). These miRNAs could be differentially expressed in platelets containing HIV, and their eventual transfer to target immune cells could participate in immune cell dysfunction as observed in InRs (64, 65). Furthermore, αIIb/β3 mRNA is a platelet-specific transcript conserved in circulating platelets throughout their life span (66) and can be exploited to track the process of transfer of mRNA/miRNA of HIV-containing platelets to leukocytes. Which of these mechanisms is at work in the metabolic immunomodulation of CD4^+^ T cells by InR platelets we report remains to be determined.

Recent evidence indicates that the cellular metabolism controls both the activation and the differentiation of CD4^+^ T cells (24). Cells use two major pathways for energy generation: glycolysis and OXPHOS. After activation, metabolically quiescent naive T cells switch from OXPHOS to glycolysis, providing energy and biosynthetic precursors for cell proliferation and effector functions. We have shown here that HIV-containing platelets increased the rates of glycolysis and the contribution of glycolysis in intracellular ATP production without affecting mitochondrial respiration in CD4^+^ T cells from healthy donors. We thus suggest that platelets from InRs could impact CD4^+^ T-cell metabolism thereby promoting immunological failure in HIV-infected ART-treated patients. Such metabolism modulation would affect uninfected bystander CD4+ T cells but also HIV-infected CD4^+^ T-cell reservoirs in which viral replication is strongly impaired in CD4^+^ T cells in glucose-deprived or glutamine-deprived conditions (67–69).

The metabolic environment that favors HIV-1 infection might also contribute to the persistence of the infected cells. Different reports have indicated that among infected cells, those cells that prevent aerobic glycolysis and preserve mitochondrial integrity and function may have a survival advantage and may better resist virus-induced cell death *in vitro* (69–71). Moreover, enhanced glycolytic activity in CD4^+^ T cells was associated with T-cell activation in HIV-infected adults (72). Mitochondrial respiration is also impaired in CD4^+^ T cells from individuals positive for HIV-1 (73) and associated with cell death, CD4^+^ T-cell depletion (74), and dysfunctional T_reg_ (17).

Altogether, the data presented here advocate for a link between T cells and platelets in immunological failure. This interface may play an important role in host defense and in chronic inflammatory diseases associated with HIV infection. Agents that might inhibit this interaction, as anticipated with abciximab, may also block the formation of conjugates and inhibit oxidative metabolism in InR patients with HIV-containing platelets and thus provide a treatment for InRs, for whom no clinical treatment is available yet. Then, future immunotherapies may need to target the metabolic programs of T cells to enhance their antiviral potential.

In sum, our data demonstrate noticeable differences in the metabolic profile of T cells cocultured with HIV-containing platelets compared to T cells cocultured with HIV-negative platelets and healthy donor platelets, suggesting a shift in the bioenergetic profile of T lymphocytes toward glycolysis after contact with HIV-containing platelets and probably linked to chronic activation.

## Data Availability Statement

The datasets presented in this study can be found in online repositories. The names of the repository/repositories and accession number(s) can be found below: https://www.ncbi.nlm.nih.gov/geo/query/acc.cgi?acc=GSE106792.

## Ethics Statement

The studies involving human participants were reviewed and approved by “Comité de Protection des Personnes” (CPP) of Ile-de-France. The patients/participants provided their written informed consent to participate in this study.

## Author Contributions

Study design: AZ, FR, CC, and MB. Methodology: AZ, FR, CC, and MB. Sample resources: CC, JZ, ER, PT, and SG. Investigation: AZ, FR, CC, and MB. Formal data analysis: AZ, FR, CC, and MB. Data interpretation: AZ, FR, CC, and MB. Funding acquisition: MB. Medical validation: CC. Writing, review, editing: AZ, FR, CC, and MB. All authors contributed to the article and approved the submitted version.

## Funding

This work was supported by funds from Agence Nationale de la recherche sur le SIDA et les Hépatites (ANRS, Funding no.: AO2019-2-19401), from Fondation pour la Recherche Médicale (Équipe FRM EQU201903007830) and the fund Line Renaud-Loulou Gasté- Fondation pour la Recherche Médicale to MB, by funds from APHP 190574 IDRCB 2020-A00307-32 to CC and by funds from the DIM (Domaine d’Interet Majeur)-1-HEALTH (funding no.: 14-DIM1HEALTH-2017) to MB and CC. AZ was supported by the China Scholarship Council. Funders of the study had no role in the study design, data collection, data analysis, data interpretation, or writing of the article. 

## Conflict of Interest

The authors declare that the research was conducted in the absence of any commercial or financial relationships that could be construed as a potential conflict of interest.

## Publisher’s Note

All claims expressed in this article are solely those of the authors and do not necessarily represent those of their affiliated organizations, or those of the publisher, the editors and the reviewers. Any product that may be evaluated in this article, or claim that may be made by its manufacturer, is not guaranteed or endorsed by the publisher.

## Supplementary Material

Supplementary Table 1 List of Differentially Expressed Genes (DEG) comparing gene expression from Immunological Non-Responders (InR) versus Immunological Responders (IR) CD4^+^ T cells, obtained by GEO2R software using the public microarray dataset GSE106792 from Gene Expression Omnibus (GEO). Table shows Illumina gene entries converted in ensemble entries, gene symbols and gene titles. Gene expression is shown as Log2 fold change and -Log10 *p* value.
